# mTOR Signaling in Metabolism and Cancer

**DOI:** 10.3390/cells9102278

**Published:** 2020-10-13

**Authors:** Shile Huang

**Affiliations:** 1 Department of Biochemistry and Molecular Biology, Louisiana State University Health Sciences Center, 1501 Kings Highway, Shreveport, LA 71130-3932, USA; shuan1@lsuhsc.edu; Tel.: +1-318-675-7759; 2 Feist-Weiller Cancer Center, Louisiana State University Health Sciences Center, 1501 Kings Highway, Shreveport, LA 71130-3932, USA

**Keywords:** mTOR, PI3K, Akt, tissue regeneration, regulatory T cells, tumor, photodynamic therapy

## Abstract

The mechanistic/mammalian target of rapamycin (mTOR), a serine/threonine kinase, is a central regulator for human physiological activity. Deregulated mTOR signaling is implicated in a variety of disorders, such as cancer, obesity, diabetes, and neurodegenerative diseases. The papers published in this special issue summarize the current understanding of the mTOR pathway and its role in the regulation of tissue regeneration, regulatory T cell differentiation and function, and different types of cancer including hematologic malignancies, skin, prostate, breast, and head and neck cancer. The findings highlight that targeting the mTOR pathway is a promising strategy to fight against certain human diseases.

The mechanistic/mammalian target of rapamycin (mTOR), a serine/threonine kinase, integrates environmental cues such as hormones, growth factors, nutrients, oxygen, and energy, regulating cell growth, proliferation, survival, motility and differentiation as well as metabolism (reviewed in [1,2]). Evidence has demonstrated that deregulated mTOR signaling is implicated in a variety of disorders, such as cancer, obesity, diabetes, and neurodegenerative diseases (reviewed in [1,2]). Current knowledge indicates that mTOR functions at least as two distinct complexes (mTORC1 and mTORC2) in mammalian cells. mTORC1 consists of mTOR, mLST8 (also termed G-protein β-subunit-like protein, GβL, a yeast homolog of LST8), raptor (regulatory-associated protein of mTOR), PRAS40 (proline-rich Akt substrate 40 kDa) and DEPTOR (DEP domain containing mTOR interacting protein), whereas mTORC2 is composed of mTOR, mLST8, rictor (rapamycin insensitive companion of mTOR), mSin1 (mammalian stress-activated protein kinase-interacting protein 1), protor (protein observed with rictor, also named PRR5, proline-rich protein 5), and DEPTOR [1,2]. mTORC1 regulates the phosphorylation or expression of p70 S6 kinase (S6K1), eukaryotic initiation factor 4E (eIF4E) binding protein 1 (4E-BP1), lipin1, ULK1 (Unc-51 like autophagy activating kinase 1), TFEB (transcription factor EB), ATF4 (activating transcription factor 4), HIF1α (hypoxia-inducible factor 1 alpha), etc., and mediates the protein synthesis/turnover, lipid synthesis and nucleotide synthesis, thus controlling cell growth, proliferation, autophagy, and metabolism (reviewed in [1,2]). mTORC2 regulates the phosphorylation/activity of Akt, serum/glucocorticoid regulated kinase (SGK), protein kinase C (PKC), etc., thereby controlling cell migration, apoptosis and metabolism (reviewed in [1,2]). These findings not only reveal the crucial role of mTOR in physiology and pathology, but also reflect the complexity of the mTOR signaling network.

The papers published in this special issue summarize the current understanding of the mTOR pathway and its role in the regulation of tissue regeneration, regulatory T cell differentiation and function, and diverse types of cancer including hematologic malignancies, skin, prostate, breast, and head and neck cancer.

Wei et al. (2019) discussed the role of mTOR signaling in the regeneration of tissues in the optic nerve, spinal cord, muscles, the liver and the intestine [3]. Activated mTOR enhances the regeneration of adult retinal ganglion cells after optic nerve injury. However, hyperactivation of mTOR in astrocytes promotes glial scar formation, leading to inhibition of spinal cord regeneration after spinal cord injury. These findings suggest that mTOR signaling exhibits opposite functions in the optic nerve and spinal cord regeneration. In mice, conditional knockout of *MTOR* or *RAPTOR* in muscle stem cells effectively inhibits activation, proliferation, and differentiation of satellite cells, impairing skeletal muscle regeneration, whereas *RICTOR* knockout in embryonic and adult satellite cells has no effect on skeletal muscle regeneration. Inhibition of mTORC1 with rapamycin inhibits the formation of nascent myofibers and the growth of regenerating myofibers during skeletal muscle regeneration. Furthermore, inhibition of mTORC1 not only suppresses the growth and proliferation of hepatocytes, but also blocks the proliferation of cholangiocytes and the formation of bipotential progenitor cells, which are essential for liver regeneration. These findings suggest that mTORC1, but not mTORC2, regulates skeletal muscle and liver regeneration. Similarly, mTORC1 is required for intestinal regeneration by controlling the proliferation and maintenance of intestinal stem cells. The development of novel drugs for tissue-specific activation or inhibition of mTOR may be beneficial to patients needing specific tissue regeneration.

Regulatory T cells (Tregs), a subset of T cells, suppress activation of the immune system and prevent autoimmune disease [4]. Chen et al. (2019) summarized the role of mTOR signaling in regulating the differentiation and function of Tregs [5]. Inhibition of mTOR with rapamycin decreases the production of effector T cells, but increases the generation and expansion of Tregs. Loss of mTORC1 signaling prevents naïve CD4+ T cells from differentiation to Th17 cells. However, disruption of either mTORC1 or mTORC2 has no effect on the differentiation of naïve CD4+ T cells into Foxp3+ Tregs. In addition, inhibition of mTORC1 attenuates the function of Tregs, while inhibition of mTORC2 increases Tregs function via promoting the activity of mTORC1, suggesting that mTORC1 and mTORC2 play opposite roles in mediating the function of Tregs. Furthermore, mTORC2 promotes the migration of Tregs to inflammatory sites. It is unclear if mTORC2 and mTORC1 are important for the expansion and migration of Tregs, respectively.

Acute lymphoblastic leukemia (ALL) is one of the aggressive hematologic malignancies that occurs in both children and adults [6]. Simioni et al. (2019) reviewed the advances in targeted therapy for ALL using mTOR inhibitors [7]. Constitutive activation of mTOR pathway is associated with deregulated production of malignant lymphoid cells and chemotherapeutic resistance in ALL. Overall, rapalogs (rapamycin, everolimus, temsirolimus) alone are primarily cytostatic, but they are synergistic with either conventional chemotherapeutic agents (doxorubicin, cyclophosphamide, dexamethasone) or other targeted therapies for ALL treatment. Treatment with dual PI3K/mTOR inhibitors (e.g., PKI-587 and BEZ235) or mTOR kinase inhibitors (e.g., AZD8055 and OSI-027) alone or in combination with chemotherapeutic agents not only inhibits cell proliferation but also induces apoptosis of ALL cells. The authors also briefly summarized clinical trials of some of these mTOR inhibitors for treatment of both T- and B-ALL.

The Warburg effect is associated with increased glycolysis, and has been implicated in chemoresistance in cancer therapy [8]. Mirabilii et al. (2020) discussed how hyperactivated mTOR, in concert with other metabolic modulators (AMPK and HIF1α) and microenvironmental stimuli, results in the acquisition of new glycolytic phenotype by directly and indirectly regulating the activity of certain key glycolytic enzymes in various hematologic malignancies [9]. For instance, in acute myeloid leukemia (AML) cells, mTOR upregulates the expression of PFKFB3 (6-phosphofructo-2-kinase/fructose-2,6-biphosphatase 3), increasing aerobic glycolysis. In chronic myeloid leukemia (CML) cells, mTOR, along with Bcr-Abl, upregulates the expression of pyruvate kinase isozymes M1/M2 (PKM1/2), enhancing aerobic glycolysis and reducing oxidative phosphorylation (OXPHOS). In acute lymphoblastic leukemia (ALL) cells, mTOR positively regulates the expression of hexokinase II, thus increasing lactate generation. The authors also discussed how these features could be targeted for therapeutic purposes.

Tan et al. (2019) reviewed genetic and epigenetic alterations of multiple genes related to the dysregulation of mTOR signaling, and discussed certain potential targets for therapeutic intervention in head and neck cancer, especially head and neck squamous cell carcinoma (HNSCC) [10]. Gain-of-function alterations (overexpression or mutations) of oncogenes (e.g., *EGFR*, *PIK3CA*, and *HRAS*) and loss-of-function mutations of tumor suppressor genes (e.g., *TP53* and *PTEN*) occur frequently in HNSCC, resulting in hyperactivation of mTOR signaling. The Cancer Genome Atlas (TCGA) database shows that mutations of *EIF4G1*, *RAC1*, *SZT2*, and *PLD1* in HNSCC also lead to aberrant mTOR signaling. Some of these mutated genes may be used as biomarkers to predict drug response. In addition, human papillomavirus (HPV) infection can activate mTOR pathway and inactivate p53 and Rb, promoting HNSCC development and progression. Accordingly, a number of clinical trials have been and are currently being conducted to evaluate the anticancer efficacy of mTOR inhibitors alone or in combination with chemotherapeutics or other kinase inhibitors.

Ayuk and Abrahamse (2019) discussed the mTOR signaling in cancer and the advances in photodynamic therapy (PDT) for cancer [11]. Mechanistically, PDT involves the application of a non-toxic photosensitizer to a specific tissue/organ, where the photosensitizer can be activated by a laser light at specific wavelengths in the presence of oxygen to generate reactive oxygen species (ROS), resulting in cell death. The effectiveness of PDT depends on the oxygen concentration, wavelength, types of photosensitizer and the genotype of tumor cells. Photosensitizers currently used include naturally occurring macrocycles (e.g., hemoglobin, vitamin B12, and chlorophyll) and tetrapyrroles (e.g., bacteriochlorins, chlorins, porphyrins, and phthalocyanines) as well as synthetic dyes. Induction of ROS by PDT inhibits the mTOR pathway. Also, PDT is synergistic with PI3K/mTOR inhibitors in cancer therapy.

Chamcheu et al. (2019) summarized the recent advances in the role of PI3K/Akt/mTOR signaling in the development and progression of skin cancers [12]. The skin comprises epidermis, dermis, and hypodermis, infiltrated by sweat glands, sensory cells, fibroblasts, macrophages, and lymphocytes. Genetic alterations or ultraviolet (UV) exposure results in the dysregulation of PI3K/Akt/mTOR pathway in melanocytes, basal cells, squamous cells, or Merkel cells, leading to melanoma, basal cell carcinoma, cutaneous squamous cell carcinoma, or Merkel cell carcinoma. The authors also discussed the current progress in preclinical and clinical studies for the development of PI3K/Akt/mTOR targeted therapies with natural phytochemicals (e.g., curcumin, epigallocatechin gallate, fisetin, resveratrol, and honokiol) and synthetic small molecule inhibitors including rapalogs and PI3K/mTOR kinase inhibitors. Some of these inhibitors are being tested in early-stage clinical trials, but their applications in the treatment of skin cancers need further testing.

Makarević et al. (2018) studied whether the inhibition of histone deacetylase (HDAC) counteracts the resistance to the mTOR inhibitor temsirolimus in a prostate cancer cell model [13]. For this, parental and temsirolimus-resistant PC3 prostate cancer cells were treated with the HDAC inhibitor valproic acid (VPA), followed by assays for tumor cell adhesion, migration, and invasion. The results indicate that treatment with temsirolimus (10 nM) inhibits the binding to human umbilical vein endothelial cells (HUVECs) or cell matrix (collagen, fibronectin, and laminin), cell migration and invasion in the parental cells, but not in the temsirolimus-resistant cells. However, treatment with VPA is able to suppress the cell adhesion, migration and invasion in both parental cells and temsirolimus-resistant cells. This is at least partly associated with a significant downregulation of integrin α5 in the resistant tumor cells. The findings suggest that inhibition of HDAC is able to block the metastatic activity in temsirolimus-resistant prostate cancer cells.

Hyperactive PI3K/AKT/mTOR signaling has been implicated in triple negative breast cancer (TNBC), which contributes to resistance to chemotherapeutic agents, including microtubule-targeting agents [14]. Eribulin mesylate, a microtubule depolymerizing agent, has been approved by the US Food and Drug Administration (FDA) to treat taxane and anthracycline refractory breast cancer. Wen et al. (2019) investigated whether eribulin enhances the anticancer activity of the mTOR inhibitor everolimus in TNBC [15]. The results indicate that treatment with eribulin, like vinblastine (a microtubule depolymerizing agent), inhibits the phosphorylation of Akt, while treatment with paclitaxel (a microtubule stabilizing agent) or cisplatin (a DNA damaging agent) has the opposite effect. Inhibition of mTORC1 with everolimus induces phosphorylation of Akt, which is blocked by eribulin. Importantly, eribulin synergizes with everolimus in reducing cell viability in vitro and inhibiting tumor growth in two orthotopic xenograft mouse models of breast cancer (MDA-MB-468 and 4T1). The findings demonstrate that combination therapy with eribulin and everolimus has a great potential to combat refractory TNBC.

## References

[1] Liu G.Y., Sabatini D.M. (2020). mTOR at the nexus of nutrition, growth, ageing and disease. Nat. Rev. Mol. Cell Biol..

[2] Mossmann D., Park S., Hall M.N. (2018). mTOR signalling and cellular metabolism are mutual determinants in cancer. Nat. Rev. Cancer.

[3] Wei X., Luo L., Chen J. (2019). Roles of mTOR Signaling in Tissue Regeneration. Cells.

[4] Raffin C., Vo L.T., Bluestone J.A. (2020). T_reg_ cell-based therapies: Challenges and perspectives. Nat. Rev. Immunol..

[5] Chen Y., Colello J., Jarjour W., Zheng S.G. (2019). Cellular Metabolic Regulation in the Differentiation and Function of Regulatory T Cells. Cells.

[6] Roberts K.G., Mullighan C.G. (2015). Genomics in acute lymphoblastic leukaemia: Insights and treatment implications. Nat. Rev. Clin. Oncol..

[7] Simioni C., Martelli A.M., Zauli G., Melloni E., Neri L.M. (2019). Targeting mTOR in Acute Lymphoblastic Leukemia. Cells.

[8] Icard P., Shulman S., Farhat D., Steyaert J.M., Alifano M., Lincet H. (2018). How the Warburg effect supports aggressiveness and drug resistance of cancer cells?. Drug Resist. Updat..

[9] Mirabilii S., Ricciardi M.R., Tafuri A. (2020). mTOR Regulation of Metabolism in Hematologic Malignancies. Cells.

[10] Tan F.H., Bai Y., Saintigny P., Darido C. (2019). mTOR Signalling in Head and Neck Cancer: Heads Up. Cells.

[11] Ayuk S.M., Abrahamse H. (2019). mTOR Signaling Pathway in Cancer Targets Photodynamic Therapy In Vitro. Cells.

[12] Chamcheu J.C., Roy T., Uddin M.B., Banang-Mbeumi S., Chamcheu R.N., Walker A.L., Liu Y.Y., Huang S. (2019). Role and Therapeutic Targeting of the PI3K/Akt/mTOR Signaling Pathway in Skin Cancer: A Review of Current Status and Future Trends on Natural and Synthetic Agents Therapy. Cells.

[13] Makarević J., Rutz J., Juengel E., Maxeiner S., Mani J., Vallo S., Tsaur I., Roos F., Chun F.K., Blaheta R.A. (2018). HDAC Inhibition Counteracts Metastatic Re-Activation of Prostate Cancer Cells Induced by Chronic mTOR Suppression. Cells.

[14] Khan M.A., Jain V.K., Rizwanullah M., Ahmad J., Jain K. (2019). PI3K/AKT/mTOR pathway inhibitors in triple-negative breast cancer: A review on drug discovery and future challenges. Drug Discov. Today.

[15] Wen W., Marcinkowski E., Luyimbazi D., Luu T., Xing Q., Yan J., Wang Y., Wu J., Guo Y., Tully D. (2019). Eribulin Synergistically Increases Anti-Tumor Activity of an mTOR Inhibitor by Inhibiting pAKT/pS6K/pS6 in Triple Negative Breast Cancer. Cells.

